# Risk Prediction Scores for Recurrence and Progression of Non-Muscle Invasive Bladder Cancer: An International Validation in Primary Tumours

**DOI:** 10.1371/journal.pone.0096849

**Published:** 2014-06-06

**Authors:** Moniek M. Vedder, Mirari Márquez, Esther W. de Bekker-Grob, Malu L. Calle, Lars Dyrskjøt, Manoils Kogevinas, Ulrika Segersten, Per-Uno Malmström, Ferran Algaba, Willemien Beukers, Torben F. Ørntoft, Ellen Zwarthoff, Francisco X. Real, Nuria Malats, Ewout W. Steyerberg

**Affiliations:** 1 Department of Public Health, Erasmus Medical Centre, Rotterdam, the Netherlands; 2 Genetic and Molecular Epidemiology Group, Spanish National Cancer Research Centre (CNIO), Madrid, Spain; 3 Systems Biology Department, University of Vic, Vic, Barcelona, Spain; 4 Department of Molecular Medicine, Aarhus University Hospital, Aarhus, Denmark; 5 Centre for Research in Environmental Epidemiology, Municipal Institute of Medical Research, Barcelona, Spain; 6 Department of Surgical Sciences, Uppsala University, Uppsala, Sweden; 7 Department of Pathology, Fundació Puigvert-University Autonomous, Barcelona, Spain; 8 Department of Pathology, Erasmus Medical Centre, Rotterdam, the Netherlands; 9 Epithelial Carcinogenesis Group, Spanish National Cancer Research Centre (CNIO), Madrid, Spain; 10 Department of Experimental and Health Sciences, Universitat Pompeu Fabra, Barcelona, Spain; Eberhard-Karls University, Germany

## Abstract

**Objective:**

We aimed to determine the validity of two risk scores for patients with non-muscle invasive bladder cancer in different European settings, in patients with primary tumours.

**Methods:**

We included 1,892 patients with primary stage Ta or T1 non-muscle invasive bladder cancer who underwent a transurethral resection in Spain (n = 973), the Netherlands (n = 639), or Denmark (n = 280). We evaluated recurrence-free survival and progression-free survival according to the European Organisation for Research and Treatment of Cancer (EORTC) and the Spanish Urological Club for Oncological Treatment (CUETO) risk scores for each patient and used the concordance index (c-index) to indicate discriminative ability.

**Results:**

The 3 cohorts were comparable according to age and sex, but patients from Denmark had a larger proportion of patients with the high stage and grade at diagnosis (p<0.01). At least one recurrence occurred in 839 (44%) patients and 258 (14%) patients had a progression during a median follow-up of 74 months. Patients from Denmark had the highest 10-year recurrence and progression rates (75% and 24%, respectively), whereas patients from Spain had the lowest rates (34% and 10%, respectively). The EORTC and CUETO risk scores both predicted progression better than recurrence with c-indices ranging from 0.72 to 0.82 while for recurrence, those ranged from 0.55 to 0.61.

**Conclusion:**

The EORTC and CUETO risk scores can reasonably predict progression, while prediction of recurrence is more difficult. New prognostic markers are needed to better predict recurrence of tumours in primary non-muscle invasive bladder cancer patients.

## Introduction

Bladder cancer is the most common malignancy of the urinary tract and a major health issue [1]. Most patients with bladder cancer are diagnosed with non-muscle invasive disease (NMIBC: stage Ta or T1) [2]. After transurethral resection (TUR), recurrence of disease occurs in 30–60% of patients and, approximately, 10–15% develop progression to muscle-invasive disease in 5-year after diagnosis [3]. Therefore, regular cystoscopy is carried out for surveillances after TUR. To better target surveillance, risk scores for recurrence and progression prediction have been developed. The best known are the European Organisation for Research and Treatment of Cancer (EORTC) [4] and the Spanish Urological Club for Oncological Treatment (CUETO) [5] risk scores; the latter focusing on BCG treated patients. Despite their potential usefulness in daily practice, few studies have externally validated these models [6]–[11] and no study focussed on primary NMIBC. In addition, since the EORTC score was based on a cohort of patients included in 7 clinical trials, the question arises whether these scores are still valid in a broader set of NMIBC patients for predictive purposes. The EORTC and CUETO scores were based on specimens evaluated by central pathologies and specialized pathologists, whereas the specimens included in the present study had been evaluated by routine pathology. In the present study, we investigated the external validity of these risk scores in patients with primary NMIBC across European centres in an everyday routine setting.

## Methods

### Study Population

We included 1,892 patients with primary NMIBC from three countries; Spain, Denmark, and the Netherlands. Patients from Spain were recruited between 1998 and 2001 from 18 general and University hospitals as part of the Spanish Bladder Cancer/EPIdemiology of Cancer of the UROthelium (EPICURO) study [12]. All centres are outlined in Appendix table S1. Patients from Denmark were selectively included based on being at higher risk of progression from patient records of the Aarhus University Hospital between 1979 and 2007 [13]. For the Netherlands, we included consecutive patients from the Erasmus MC who underwent a TUR between 1990 and 2012. Patient and tumour characteristics and data on recurrence and progression after TUR of the primary NMIBC were extracted from hospital records up till November 2012. All patients had histologically confirmed NMIBC and were treated according to the centres’ usual procedures. At the Erasmus MC in the Netherlands, follow-up of patients was according to the EAU guidelines at the time, and risk-adapted according to the EORTC risk scores outcome. At the Aarhus University Hospital in Denmark, the common follow-up strategy for all patients was every three months. In Spain, protocols for the follow-up of bladder cancer patients were developed within each centre. For non-muscle invasive bladder cancers, follow-up for these patients consisted of bladder endoscopy every three months the first year, every six months the second year and then annually bladder endoscopy to complete five years of monitoring. White light cystoscopy was used in all centres participating in our study.

Disease progression was defined as cystoscopically detected tumour relapse with histological confirmation at tumour stage T2 or higher (progression to a muscle invasive tumour stage); it was assumed that a tumour progression always precedes death because of cancer. Patients that died because of bladder cancer without a progression were recorded as having had a progression at the time of death. Recurrence was defined as cystoscopically detected tumour relapse with histological confirmation. Data from the 3 cohorts were harmonized, anonymized, and combined in one data set for statistical analyses, stratified by cohort.

All Danish and Spanish patients gave their written informed consent, and the study was approved by the Central Denmark Region Committees on Biomedical Research Ethics (1994/2920) and by the Ethics Committees of each Spanish participating centre and the Institutional Review Board of the U.S. National Cancer Institute, NIH, USA. This observational study was exempted from formal ethical approval in the Netherlands. All data is anonymized before being used in this study.

### Risk Scores

The EORTC scores for recurrence and progression were based on data from 2,596 patients diagnosed with Ta/T1 tumours from seven EORTC trials [4]. A limitation of the EORTC scores was the low number of patients treated with bacillus Calmette Guérin (BCG). Therefore, the CUETO group developed a scoring model in 1,062 BCG-treated patients [5]. The EORTC score incorporated the number of tumours (single, 2–7 or ≥8), tumour size (<3 cm or ≥3 cm), prior recurrence rate (primary, ≤1 recurrence/year, >1 recurrence/year), T stage (Ta or T1), concomitant carcinoma in situ (yes/no), and grade (1, 2, or 3). The CUETO model incorporated gender, age (<60, 60–70, >70 years), recurrent tumour (yes/no), number of tumours (≤3 or >3), T stage (Ta or T1), concomitant carcinoma in situ (yes/no), and grade (1, 2, or 3).

### Validation

For all patients, we calculated risks for recurrence and progression according to the EORTC and CUETO scores based on the primary tumour. Standard pathologic procedures were followed in each cohort. Tumour grade was scored according to the 1973 system, and pathological stage was according to the 2002 staging system. The presence of concomitant carcinoma in situ was incomplete (CIS, n = 990, 52% missing), as well as data on the number of tumours (n = 346, 18% missing). We used a multiple imputation strategy [14] resulting in five sets of complete data to compute risk scores. We subsequently averaged these risk scores for each patient. Patient scores were then categorized into four risk groups, i.e. low, intermediate low, intermediate high, and high risk for recurrence or progression, as originally specified for the EORTC and CUETO scores. The two highest risk groups were combined because of low numbers. Observed recurrence-free survival (RFS) and progression-free survival (PFS) were calculated from the date of TUR of the primary tumour. An event for RFS was defined as recurrence or progression, if progression occurred as the first event during follow-up. Follow-up was censored at either the last date of follow-up, the date of death, or 120 months. We used standard Kaplan–Meier plots to visualize recurrence and progression patterns in relation to risk groups. This cause-specific analysis was not adjusted for the competing risk of death before recurrence or progression, since we focused on the discriminative ability of the 2 risk scores (quantified by a concordance measure, c-index) [15]. We conducted subgroup analyses for patients receiving only BCG treatment after TUR. Furthermore, we refitted the scores with a Cox regression analysis stratified by cohort by recalculating risk scores with EORTC and CUETO coefficients based on our data, to obtain further insight in the validity of the scores. We used likelihood ratio statistics to determine the statistical significance of predictors. For comparability with the original EORTC and CUETO scores, we scaled the refitted regression coefficients by the inverse of the Cox regression coefficient for the original scores in our data. For example, the refitted score for T1 vs Ta in the EORTC model for recurrence was calculated as: multivariable coefficient for T1 vs Ta*1/(coefficient for EORTC score for recurrence). SPSS (version 20.0, SPSS Inc, Chicago, Illinois, USA) and R (Version R-2.15.2 for Windows, http://www.r-project.org/) were used for data analysis.

## Results

### Study Population

We included 1,892 patients; 280 patients from Denmark, 639 from the Netherlands, and 973 from Spain. During 10 years of follow-up, 209 (11%) patients died before a recurrence occurred, 839 (44%) patients had a recurrence and 258 (14%) a progression. Median follow-up for those without recurrence was 74 months. There were 98 patients (N = 90 from the Netherlands, N = 8 from Denmark) without follow-up because of loss to follow-up immediately after TUR. CIS (yes/no) and number of tumours was imputed in patients with missing data, based on 902 patients with information on CIS and 1546 patients with information on the number of tumours, as well as complete information on tumour stage, grade, and size, and progression and recurrence free survival (time and yes/no). The mean age was 66 years and the majority was male (Table 1). We do not present totals over all cohorts because of the substantial differences in settings between cohorts. Danish patients presented a larger proportion of patients with high stage and grade (P<0.01), and relatively more recurrences and progressions. The distribution of patients over the risk groups is shown in table 2.

**Table 1:** Patient characteristics of 1,892 patients with non-muscle invasive bladder cancer in the participating cohorts.*

		Denmark (n = 280)		Netherlands (n = 639)		Spain (n = 973)	
**Age**	Mean (SD)	66.4	10.2	65.3	12.4	65.7	10.0
**Gender**	Male	219	78.2%	503	78.7%	850	87.4%
**Stage**	Ta	177	63.2%	432	67.6%	818	84.1%
	T1	103	36.8%	207	32.4%	155	15.9%
**Grade**	G1	78	27.9%	189	29.6%	419	43.1%
	G2	83	29.6%	283	44.3%	327	33.6%
	G3	119	42.5%	167	26.1%	227	23.3%
**Carcinoma in situ**	CIS	89	31.8%	52	8.1%	0	0.0%
	No CIS	189	67.5%	572	89.5%	0	0.0%
	Missing	2	0.7%	15	2.4%	973	100.0%
**Tumour size**	<3 cm	175	62.5%	238	37.2%	564	58.0%
	≥3 cm	73	26.1%	114	17.9%	133	13.6%
	Missing	32	11.4%	287	44.9%	276	28.4%
**Number of tumours**	1	82	29.3%	349	54.6%	647	66.5%
	>1	13	4.6%	178	27.9%	277	28.5%
	Missing	185	66.1%	112	17.5%	49	5.0%
**Treatment**	TUR alone	227	81.0%	140	21.9%	404	41.5%
	TUR+BCG	52	18.6%	108	16.9%	289	29.7%
	TUR+Chemo	0	0.0%	80	12.5%	212	21.8%
	TUR+Chemo+BCG	1	0.4%	29	4.5%	51	5.2%
	Other	0	0.0%	5	0.8%	17	1.7%
	Missing	0	0.0%	277	43.4%	0	0.0%
**Status tumour** **	Recurrence	209	74.6%	303	47.4%	327	33.6%
	Progression	66	23.6%	99	15.5%	93	9.6%
**Vital status** **	Alive	72	25.7%	321	50.2%	700	71.9%
	Cancer death	12	4.3%	51	8.0%	62	6.4%
	Other death	7	2.5%	90	14.1%	211	21.7%
	Missing	189	67.5%	177	27.7%	0	0.0%

*Numbers are totals (%), unless stated otherwise.

**At the end of follow-up.

**Table 2:** Distribution of patients over the risk groups for all patients (n = 1892) and BCG treated patients (n = 449).

Risk category	CUETO				EORTC			
	Recurrence		Progression		Recurrence		Progression	
**All patients (N = 1892)**								
Low risk	1195	(63.2%)	1289	(68.1%)	383	(20.2%)	346	(18.3%)
Intermediate risk	421	(22.3%)	135	(7.1%)	1099	(58.1%)	929	(49.1%)
High risk*	276	(14.6%)	468	(24.7%)	410	(21.7%)	617	(32.6%)
**BCG (N = 449)**								
Low risk	226	(50.3%)	241	(53.7%)	48	(10.7%)	30	(6.7%)
Intermediate risk	136	(30.3%)	36	(8.0%)	257	(57.2%)	197	(43.9%)
High risk*	87	(19.4%)	172	(38.3%)	144	(32.1%)	222	(49.4%)

*The high risk group is the combined group from intermediate-high and high-risk EORTC and CUETO scores, because of low patient numbers.

### Validation

**Figure 1:**
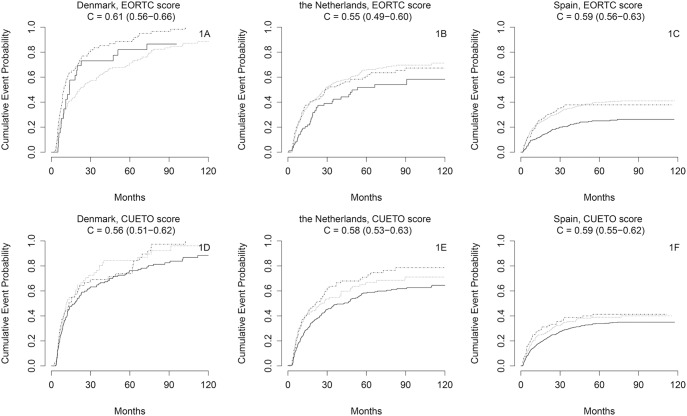
A–F. Kaplan-Meier estimates of recurrence of bladder cancer in a ten-year period from transurethral resection of a non-muscle invasive bladder tumour. Full line: low risk patients, dotted line: intermediate risk patients, dashed line: high risk patients. Number of patients per country: Denmark n = 280; The Netherlands n = 639; Spain n = 973.

**Figure 2:**
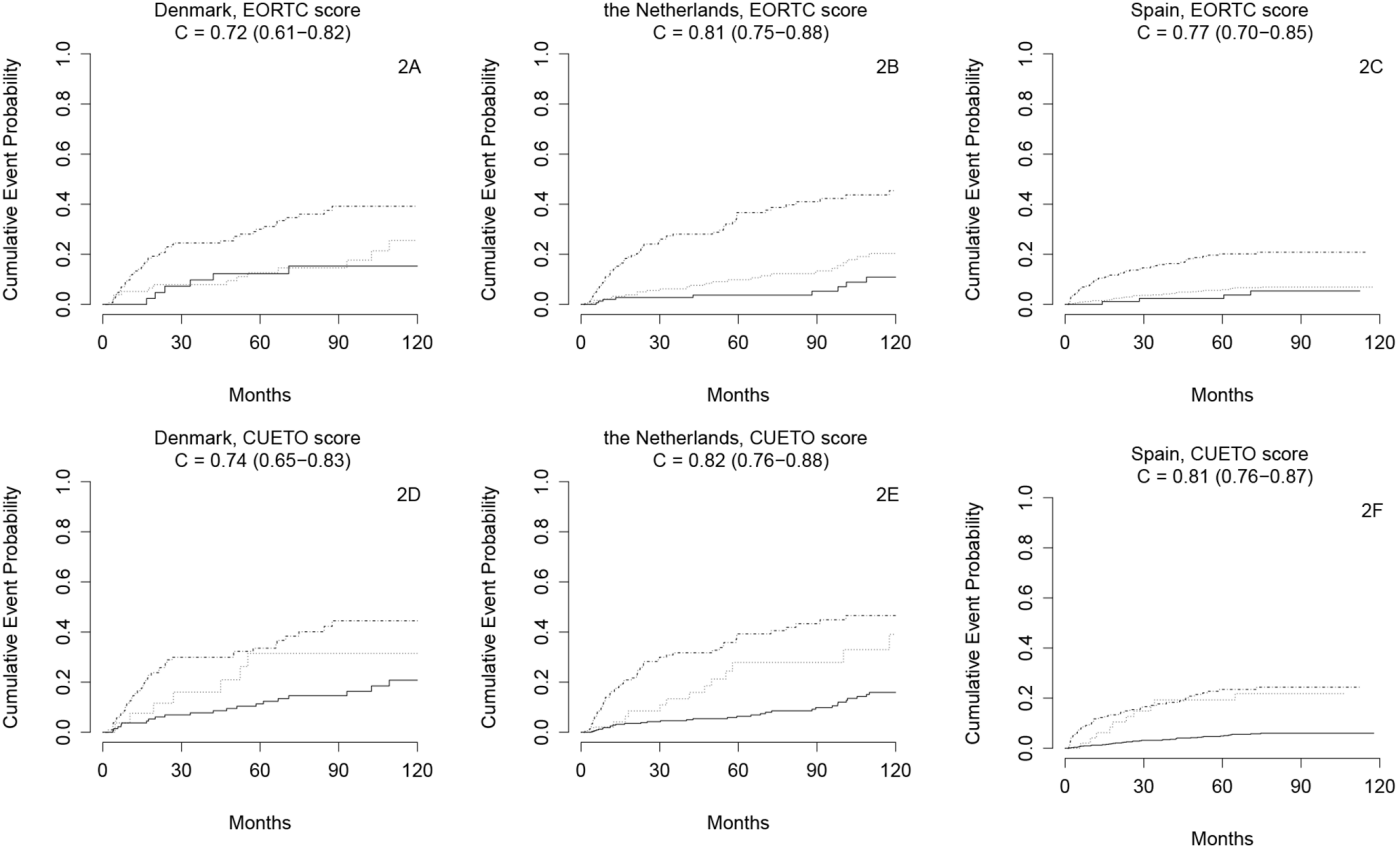
A–F. Kaplan-Meier estimates of progression of bladder cancer in a ten-year period from transurethral resection of a non-muscle invasive bladder tumour. Full line: low risk patients, dotted line: intermediate risk patients, dashed line: high risk patients. Number of patients per country: Denmark n = 280; The Netherlands n = 639; Spain n = 973.

The EORTC score could not well separate low risk from high risk patients with respect to disease recurrence (Figures 1a–c, c-indices 0.55 to 0.61). Discrimination was somewhat better for progression (Figures 2a–c, c-indices 0.72 to 0.81). The CUETO score had a similar performance (figures 1d–f and 2d–f). Subgroup analyses in patients receiving BCG treatment (n = 449) showed poorer results (Figures S1a–f and S2a–f).

When we refitted the EORTC score for recurrence in Cox regression models, the prognostic effects of multiple tumours, tumour size, CIS and tumour grade were largely confirmed, but T1 tumours had no increased risk over Ta tumours (Results not shown). For progression, tumour size and CIS were less predictive than in the original EORTC score, while the effect for grade was stronger. For the CUETO score, gender was confirmed to be predictive of recurrence. While older age was not predictive of recurrence, we confirmed its value for predicting progression in the refitted CUETO score (p<0.01).

## Discussion

The EORTC risk tables have become a standard of care with their inclusion in European guidelines [2]. The CUETO risk model was developed more recently, with a focus on patients treated with BCG. External validation of a prognostic model on a new dataset is crucial to assess its generalizability [16]. In our study, the EORTC and CUETO risk scores showed only modest discriminative ability for the recurrence of NMIBC, with c-indices of, at most, 0.61. Prediction of progression was better with c-indices ranging from 0.72 to 0.82. Our findings were consistent in the cohorts from Denmark, Spain and the Netherlands, and are in line with another external validation of the EORTC risk score [6] and with validation in primary bladder cancer cases [11].

Remarkably, the CUETO score was specifically developed for patients treated with BCG, but discriminated better in the overall population than in the selected BCG population. BCG treatment, which has become a common treatment to manage intermediate- and high-risk NMIBC [17], was used in 449 patients, of over 50% at low risk of recurrence and progression according to the CUETO risk scores. For the EORTC risk scores, we noted that BCG treatment was usually administered to higher risk patients with a relatively narrow distribution of risk scores. This homogeneity in risk may partly explain the poor discriminative ability of the scores in those treated with BCG [18]. More research in this specific group of patients needs to be done, also because of the lack of statistical power due to low numbers of BCG patients in the current study.

In the original study that presented the EORTC risk scores, prior recurrence rate was an important prognostic factor for both recurrence and progression [4]. In the clinical setting, we need to establish a surveillance plan already after TUR for the primary tumour. Therefore, it is of great importance that the EORTC risk score has predictive value also for these patients, who have not had one or multiple recurrences. We found that predicting recurrence was very difficult for primary tumours. The heterogeneity in recurrence risk becomes better known once one or more recurrences have been observed [19].

A possible explanation for the poor performance of the risk scores for the prediction of recurrence outside controlled trials is interobserver variability in bladder cancer staging and grading by pathologists. To partly overcome these issues, new methods for bladder cancer pathology have been introduced in 1998 [20] and 2004 [21]. The 1998 method has been shown to be an improvement over the 1973 method [22], which was used for our patients.

The poor predictability of recurrence may also relate to other factors, unrelated to the (observed) pathology of the disease. For example, detection of all primary tumours may be difficult at primary tumour presentation. Tumour tissue may be left behind, falsely leading to classification as a recurrent tumour. The quality of the TUR may be important but it could not be considered in our evaluation. Moreover, detection policies may vary between urologists with respect to surveillance intervals and treatment modalities (e.g. TUR vs ablation). Progression is a more robust end point, which may partly explain its better predictability with the EORTC and CUETO scores.

The retrospective analysis is a limitation of this study, and explains the presence of missing values in important variables such as CIS and tumour size. We used multiple imputation, which has been shown to be a reliable method to handle missing data [23] [23]. We had no detailed information on treatments and surveillance policies, which may have changed over time. The treatment modalities may have led to a dilution of differences between the risk groups. On the other hand, a real life situation was considered with respect to the standard care of urologists. We furthermore note that a selected group of high risk patients was included from Denmark, which can be explained by the fact that patients originated form a specialised university medical centre. However, patients from Spain were a representative sample from standard primary NMIBC population in that country, and patients from the Netherlands, though originating from an academic centre, were similar to the general Dutch primary NMIBC patient population [24].

It is clear that the EORTC and CUETO scores need further improvement. Several markers have shown promising results, such as FGFR3 and Ki67, which improved c-indices for prediction of progression from 0.75 to 0.82 in one study [8]. Various other promising molecular and germline markers are available, which need further rigorous evaluation for their usefulness to predict recurrence and progression [25]–[26]. Future risk scores will again need external validation, considering discrimination and other aspects of predictive performance, such as calibration (correspondence between observed and predicted risks) and clinical usefulness (ability to make better decisions) [27]–[29].

We conclude that the discriminatory ability of currently available risk scores is poor for recurrence and moderate for progression in primary NMIBC. Since successful discrimination of low and high risk patients is essential to the right intensity of bladder cancer surveillance, new risk markers are urgently needed to improve risk classification in NMIBC patients.

## Supporting Information

Figure S1 **A–F. Kaplan-Meier estimates of recurrence of bladder cancer in a ten-year period from transurethral resection of a bladder tumour for patients with non-muscle invasive bladder cancer treated with BCG.** Full line: low risk patients, dotted line: intermediate risk patients, dashed line: high risk patients. Number of patients per country: Denmark n = 52; The Netherlands n = 108; Spain n = 289.
(TIF)

Figure S2 **A–F. Kaplan-Meier estimates of progression of bladder cancer in a ten-year period from transurethral resection of a bladder tumour for patients with non-muscle invasive bladder cancer treated with BCG.** Full line: low risk patients, dotted line: intermediate risk patients, dashed line: high risk patients. Number of patients per country: Denmark n = 52; The Netherlands n = 108; Spain n = 289.
(TIF)

Table S1 **Centres and members of the Spanish study group.**
(DOC)
