# Supplementary figures and images for: Risk Prediction Scores for Recurrence and Progression of Non-Muscle Invasive Bladder Cancer: An International Validation in Primary Tumours
Source: PLoS One. 2014 Jun 6;9(6):e96849. doi: 10.1371/journal.pone.0096849 (PMC4048166; doi:10.1371/journal.pone.0096849)

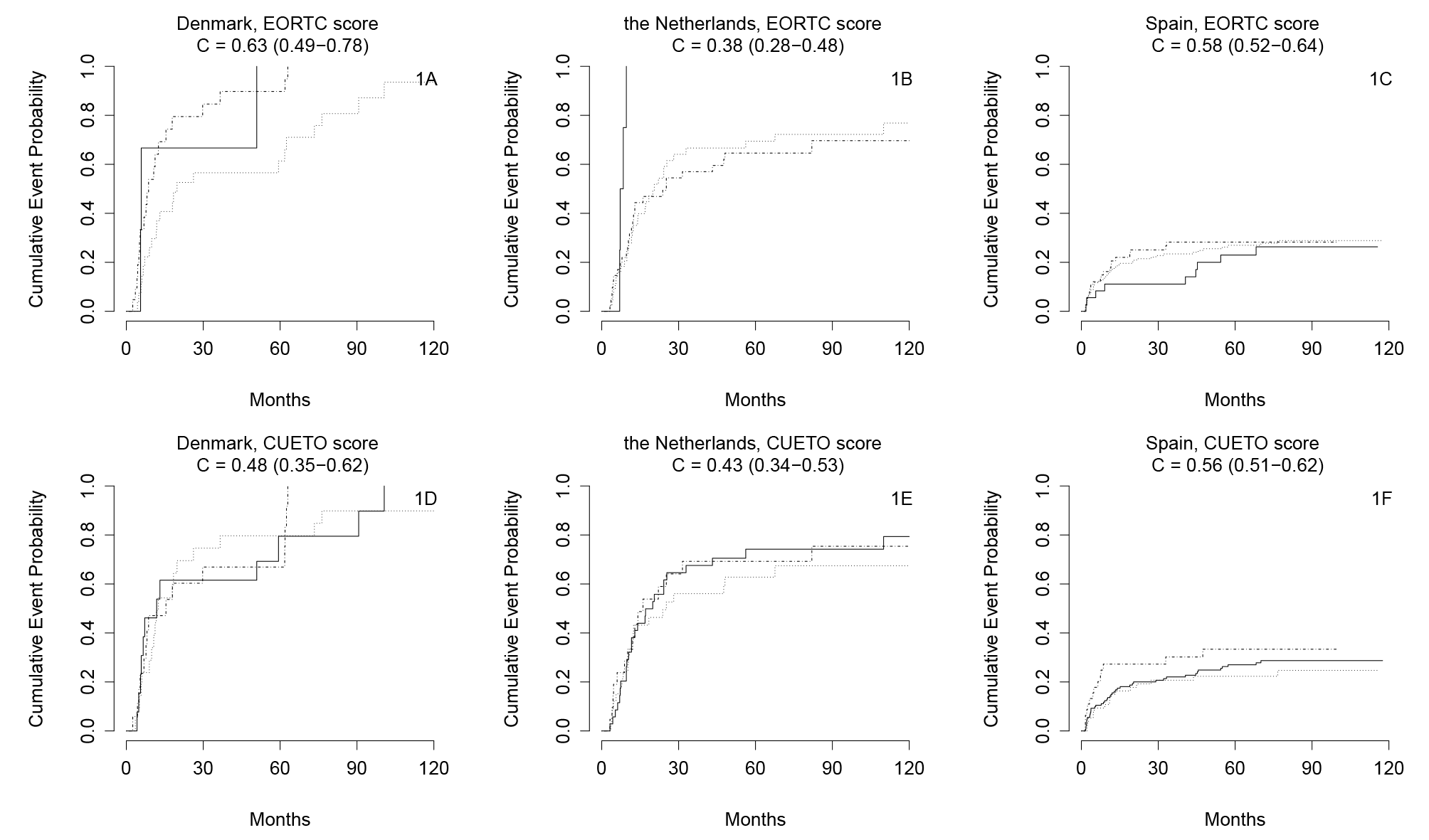

Supplement: Figure S1 — A–F. Kaplan-Meier estimates of recurrence of bladder cancer in a ten-year period from transurethral resection of a bladder tumour for patients with non-muscle invasive bladder cancer treated with BCG. Full line: low risk patients, dotted line: intermediate risk patients, dashed line: high risk patients. Number of patients per country: Denmark n = 52; The Netherlands n = 108; Spain n = 289. (TIF) [file pone.0096849.s001.tif]

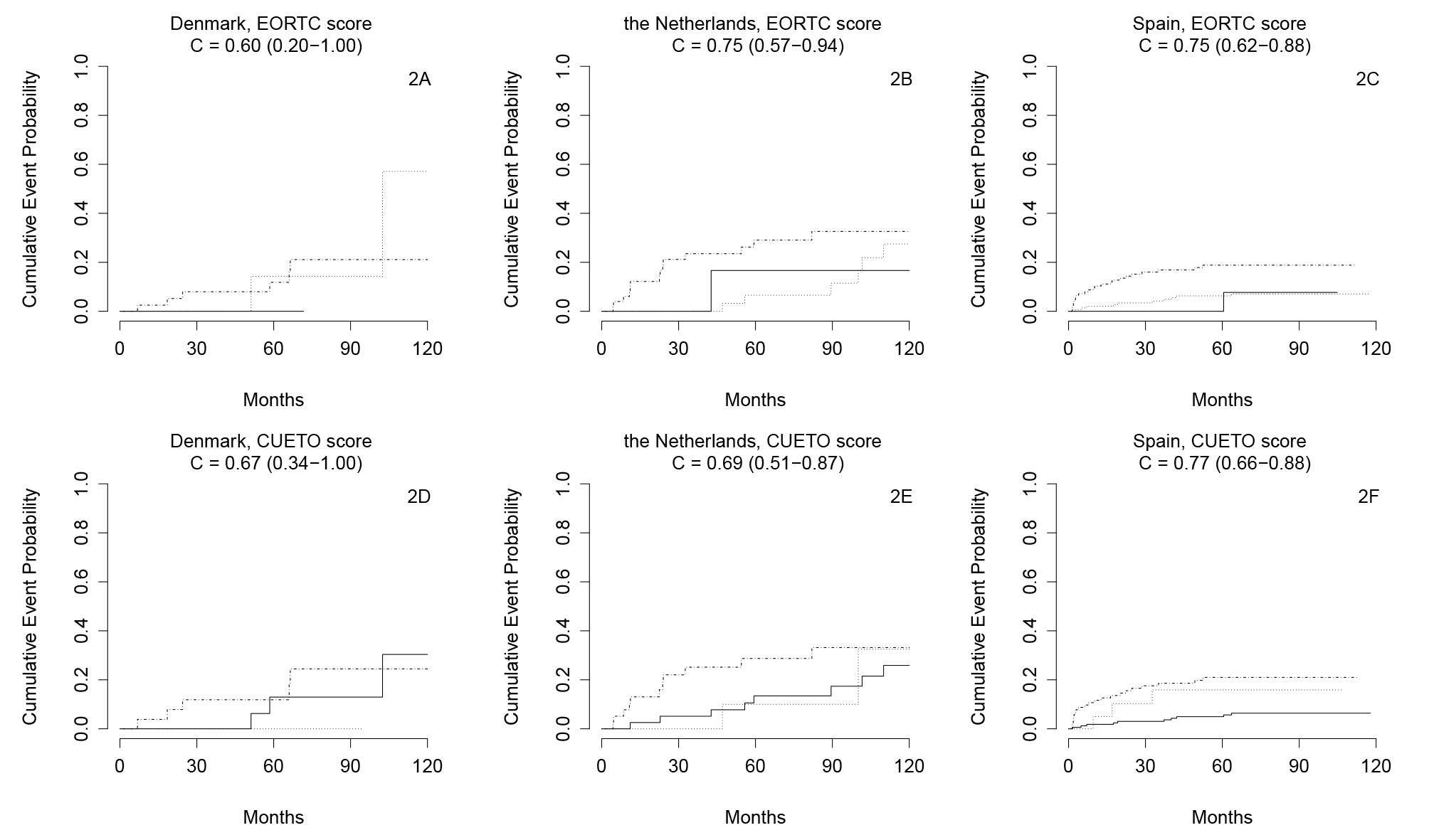

Supplement: Figure S2 — A–F. Kaplan-Meier estimates of progression of bladder cancer in a ten-year period from transurethral resection of a bladder tumour for patients with non-muscle invasive bladder cancer treated with BCG. Full line: low risk patients, dotted line: intermediate risk patients, dashed line: high risk patients. Number of patients per country: Denmark n = 52; The Netherlands n = 108; Spain n = 289. (TIF) [file pone.0096849.s002.tif]
