# Supplementary material for: Risk Prediction Scores for Recurrence and Progression of Non-Muscle Invasive Bladder Cancer: An International Validation in Primary Tumours
Source: PLoS One. 2014 Jun 6;9(6):e96849. doi: 10.1371/journal.pone.0096849 (PMC4048166; doi:10.1371/journal.pone.0096849)
Supplement: Table S1 — Centres and members of the Spanish study group. (DOC) [file pone.0096849.s003.doc]

# SUPPORTING INFORMATION

Table S1. Centres and members of the Spanish study group

| Area | Center | N |
| --- | --- | --- |
| Barcelona | Institut Municipal d’Investigació Mèdica, Universitat Pompeu Fabra (coordinating centre) |  |
| Barcelona | Hospital del Mar (Barcelona) | 88 |
| Barcelona | Hospital Germans Tries i Pujol (Badalona, Barcelona) | 101 |
| Barcelona | Hospital de Sant Boi (Sant Boi, Barcelona) | 27 |
| Barcelona | Centre Hospitalari Parc Taulı́ (Sabadell, Barcelona) | 95 |
| Barcelona | Centre Hospitalari i Cardiològic (Manresa, Barcelona) | 64 |
| Tenerife | Hospital Universitario (La Laguna, Tenerife) | 42 |
| Tenerife | Hospital La Candelaria (Santa Cruz, Tenerife) | 107 |
| Alicante | Hospital General de Elche (Elche, Alicante) | 84 |
| Asturias | Hospital Monte Naranco (Oviedo, Asturias) | 2 |
| Asturias | Hospital San Agustin (Aviles, Asturias) | 86 |
| Asturias | Hospital Central Covadonga (Oviedo, Asturias) | 71 |
| Asturias | Hospital Central General (Oviedo, Asturias) | 26 |
| Asturias | Hospital de Cabueñes (Gijón, Asturias) | 63 |
| Asturias | Hospital de Jove (Gijón, Asturias) | 35 |
| Asturias | Hospital de Cruz Roja (Gijón, Asturias) | 27 |
| Asturias | Hospital Alvarez-Buylla (Mieres, Asturias) | 21 |
| Asturias | Hospital Jarrio (Coaña, Asturias) | 24 |
| Asturias | Hospital Carmen y Severo Ochoa (Cangas, Asturias) | 10 |
